# Critical aggregation concentration for the formation of early Amyloid-β (1–42) oligomers

**DOI:** 10.1038/s41598-018-19961-3

**Published:** 2018-01-29

**Authors:** Mercedes Novo, Sonia Freire, Wajih Al-Soufi

**Affiliations:** Department of Physical Chemistry, Faculty of Science, University of Santiago de Compostela, E-27002 Lugo, Spain

## Abstract

The oligomers formed during the early steps of amyloid aggregation are thought to be responsible for the neurotoxic damage associated with Alzheimer’s disease. It is therefore of great interest to characterize this early aggregation process and the aggregates formed, especially for the most significant peptide in amyloid fibrils, Amyloid-β(1–42) (Aβ42). For this purpose, we directly monitored the changes in size and concentration of initially monomeric Aβ42 samples, using Fluorescence Correlation Spectroscopy. We found that Aβ42 undergoes aggregation only when the amount of amyloid monomers exceeds the critical aggregation concentration (*cac*) of about 90 nM. This spontaneous, cooperative process resembles surfactants self-assembly and yields stable micelle-like oligomers whose size (≈50 monomers, *R*_*h*_ ≈ 7–11 nm) and elongated shape are independent of incubation time and peptide concentration. These findings reveal essential features of *in vitro* amyloid aggregation, which may illuminate the complex *in vivo* process.

## Introduction

Alzheimer’s disease (AD) is a neurodegenerative disease characterized by the presence of Amyloid-β plaques in the brain. Although the causal relationship between these protein fibrillar aggregates and the neurodegenerative disease has not been established yet, the ‘amyloid hypothesis’, that accumulation and aggregation of amyloid-β peptide initiates a cascade of neurodegenerative events, has been widely accepted^1–3^. Impairment of Amyloid-β clearance in AD patients seems to be the main cause for accumulation of the peptide^4,5^. It is thought that the neurotoxic species that trigger the amyloid cascade leading to neurodegeneration are early non-fibrillar aggregates, which may also be the precursors of the amyloid fibrils^2,6–8^. The dominant peptides in amyloid fibrils are Amyloid-β(1–42) (Aβ42) and Amyloid-β(1–40) (Aβ40), with Aβ42 being the more fibrillogenic of the two, with a much stronger tendency to aggregate^9–11^.

There is ample literature on the mechanism underlying amyloid fibril formation^12,13^. Most kinetic studies agree on a complex nucleation-growth mechanism, where the differences in the microscopic rates and in the relevance of secondary nucleation processes determine the degree of aggregation and can account for the differences between Aβ40 and Aβ42^11,14^. For such nucleation-dependent processes, a critical aggregation concentration (*cac*) is predicted, above which aggregation takes place^10^. For Aβ40 the formation of micelle-like intermediates was reported, with a critical concentration in the micromolar range^15–17^, whereas recent studies have found nanomolar *cac* values for both Aβ40 and Aβ42^18–20^. The latter values fit better with the reported physiological concentrations of Aβ in the picomolar to nanomolar range which may be locally higher due to accumulation or impairment of clearance^4,5,18,21,22^. A possible reason for the discrepancy in the cac values may be the strong adsorption of Amyloids Aβ40 and Aβ42 to interfaces^23^, which can lead to great differences between the nominal and the real concentrations of amyloid in solution. Furthermore, adsorption on surfaces may also mediate the aggregation of amyloid at nanomolar concentration as reported in a recent study^24^.

Our main objective is to present a study of the early aggregation of Ab42 *in vitro* in absence of disaggregating agents applying a technique which allows us to follow directly the number of monomers and aggregates, their size and form and the total available Ab42 concentration. For this aim, we apply Fluorescence Correlation Spectroscopy (FCS), a technique which yields information about the size and concentration of free diffusing particles in solution. FCS data were reported for the shorter amyloid Aβ40; however, these raw data were not corrected for the artefacts inherent in the technique and were only qualitatively analysed^15^. Here we study the early aggregation process of the more relevant Aβ42 in order to derive a quantitative estimate of the critical concentration, *cac*, for the oligomer formation and to characterize the aggregates formed. Moreover, we aim to establish whether the critical concentration refers to the formation of fibrils or early non-fibrillar aggregates.

## Results and Discussion

### Samples of Aβ42

In order to obtain homogenous samples and reproducible measurements of Aβ42 aggregation, it is crucial to start with monomeric material and to control carefully the sample preparation procedure. As described in detail in the Methods section, first we obtained the monomeric peptides, following established disaggregation protocols. Then we took great care to eliminate any residual disaggregation agents in the final samples and followed a very methodical sample preparation protocol in order to ensure that the results were as reproducible as possible. The samples used for the FCS measurements contained as fluorescent probe a fixed concentration of fluorescently labelled Aβ42 (denoted by Aβ*), and different concentrations of unlabelled Aβ42 (denoted by Aβ°) in a physiological buffer.

### Diffusional properties of Aβ42 at different concentrations and incubation times

We studied the early aggregation of Aβ42 using Fluorescence Correlation Spectroscopy (FCS), an established technique which measures the changes in the diffusional properties of fluorescent probes in a confocal volume at the single molecule level^25^. Fluorescence correlation (FCS) curves of samples containing labelled and unlabelled Aβ42 were measured as a function of incubation time (within the first 2–3 hours after preparation) and total Aβ42 concentration. The analysis of the FCS curves then yields the diffusion correlation times of monomers and aggregates and their contributions to the diffusion term of the correlation curve, which are related to the size and concentration of these species. In order to be able to extract quantitative information about the aggregation process and the size and conformation of the aggregates, we developed a model for the composition and properties of the samples based on a few reasonable assumptions and applied it in a detailed data analysis (see SI).

### Monomeric Aβ42

In order to check that the starting peptides were properly disaggregated and to characterize the monomeric Aβ42, we first measured samples containing only labelled Aβ42, Aβ*, at low concentration. The FCS power series of these samples (see Supplementary Figure S1) show a single fluorescent species with a mean diffusion time through the sample volume of 153.6 ± 0.2 μs (Supplementary Equation S1). From this time and the known dimensions of the sample volume, we estimated a translational diffusion coefficient of *D* = (1.35 ± 0.03) × 10^−10^ m^2^ s^−1^ (see Methods section). This value is in agreement with the series of diffusion coefficients obtained by Danielsson *et al*. for monomeric Aβ40 and several fragments of this peptide using PFG-NMR, and shows that, under our starting conditions, Aβ42 is monomeric^26^. Moreover, our value for the diffusion coefficient of monomeric Aβ42 fits well with the molar mass dependency *D*~*M*^−*ν*^ with ν = 0.44 given by these authors, who interpreted this exponent as an indication that these small peptides in their native form have a random coil conformation with some structured regions. Molecular dynamics simulations combined with NMR data lead to similar conclusions for monomeric Aβ42 in aqueous solution^27^. Using the Stokes-Einstein equation, we estimate a hydrodynamic radius of 1.8 nm for Aβ42, which agrees quite well with that obtained in a recent study^28^.

### Aggregation of Aβ42 – Influence of incubation time

**Figure 1:**
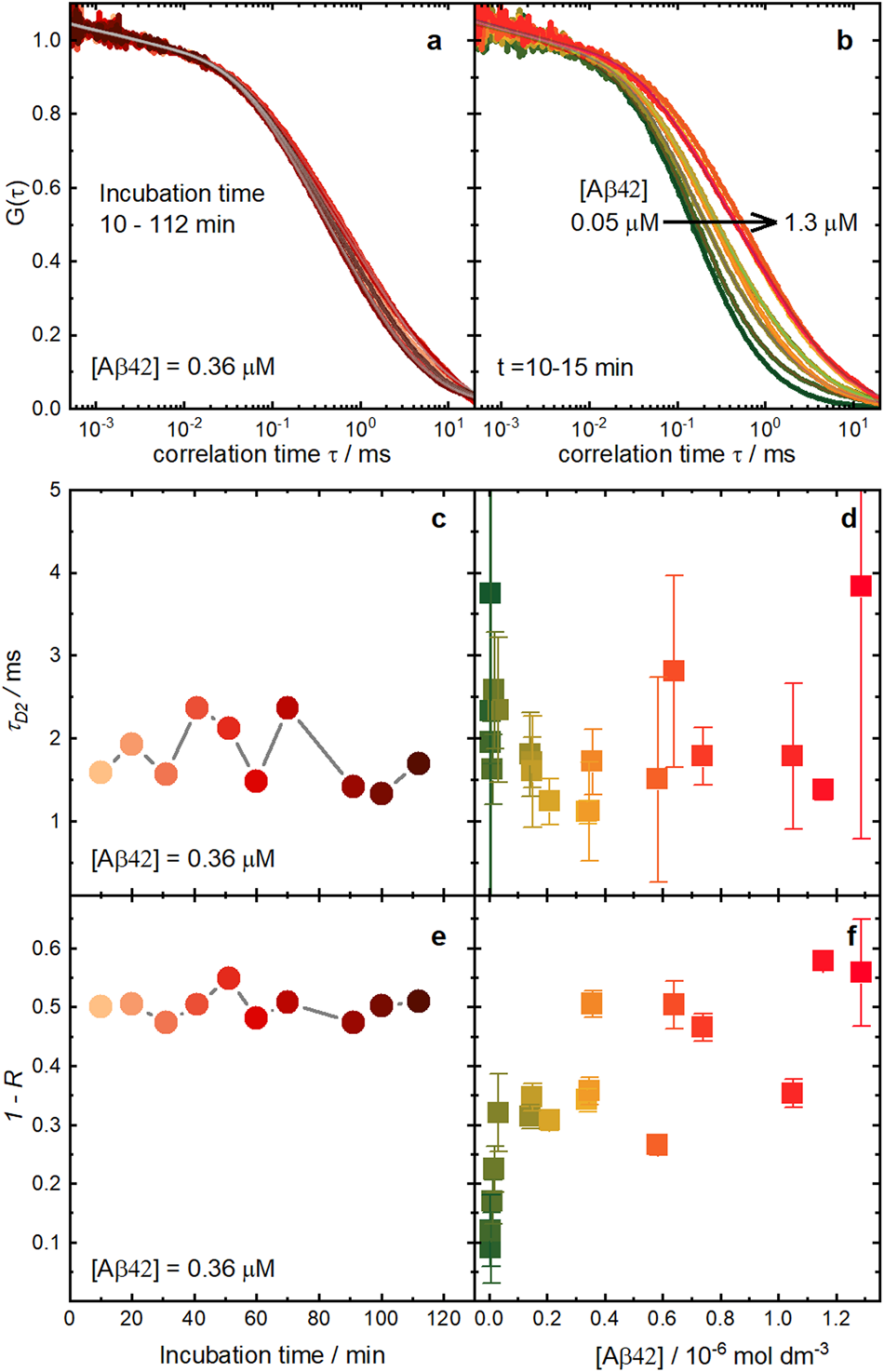
Dependence of Aβ42 amyloid aggregation on incubation time and Aβ42 concentration. Examples of FCS measurements of samples with labelled Aβ42, Aβ*, mixed with different concentrations of unlabelled Aβ42, Aβ°: (**a**) experimental and fitted correlation curves at different incubation times for a fixed total amyloid concentration in solution of 0.36 μM; (**b**) experimental and fitted FCS curves for different Aβ42 concentrations at the same short incubation time of 10–15 min; (**c**) diffusion times of the aggregates (*τ*_*D2*_) determined from the FCS curves of panel *a* against incubation time; (**d**) mean diffusion times of the aggregates (*τ*_*D2*_) obtained from the FCS curves measured at different incubation times for each sample against total Aβ42 concentration; (**e**) contribution of the aggregates (1-*R*) to the diffusion term for the FCS curves of panel *a* against incubation time; (**f**) mean contribution of the aggregates (1-*R*) obtained from the FCS curves measured at different incubation times for each sample against total Aβ42 concentration. The error bars in panels d and f indicate the standard deviation of the values measured for each sample at different incubation times. The Aβ42 concentration refers to the experimentally determined real concentrations in solution.

In order to study the early aggregation, we prepared samples with different total concentrations of disaggregated Aβ42, each sample with a fixed concentration of labelled Aβ42, Aβ*, but different concentrations of unlabelled Aβ42, Aβ° (Fig. 1a and b). Then, under the same conditions as before, we recorded FCS curves of the samples at different incubation times from 10 min up to 180 min.

All correlation curves are well fitted with two diffusion correlation times (Supplementary Equation S2, Fig. 1a and b), indicating that particles of two significantly different sizes are present in the solution. The shorter of the two diffusion times, *τ*_*D1*_, coincides with the one we obtained before for monomeric Aβ42. The second time, *τ*_*D2*_, is about 10 times longer and we attribute it to the much bigger and thus slower diffusing Aβ42 aggregates present in the sample.

Surprisingly, this second diffusion time, corresponding to Aβ42 aggregates, is already present at the shortest incubation times, 5–10 min after the preparation of the samples, independently of the Aβ42 concentration. The value of this diffusion time fluctuates in the range from 1 ms to 3 ms but shows no systematic dependence on the incubation time (Fig. 1c). These fluctuations are due to the single molecule character of FCS, which in each measurement samples a relatively small number of aggregates taken from the broad distribution of aggregate sizes. Additionally, for each Aβ42 concentration, the contribution of the aggregates to the FCS curves (factor 1-*R* in Supplementary Equation S2) is constant over the incubation time (Fig. 1e), indicating that the proportion of aggregates present in each sample is also independent of the time elapsed after sample preparation. We will confirm this observation below in a quantitative analysis, correcting the dependence of *R* on the brightness of the diffusing species.

So far, these experimental findings indicate that the observed aggregates are formed immediately (<10 min) after dissolving monomeric Aβ42 in the buffer, confirming a very short or inexistent lag time for the aggregation of Aβ42 in the concentration range under study, as already suggested in the literature^10,19,29^. Furthermore, they show that the early aggregation of Aβ42 leads to the formation of stable oligomers whose mean size and concentration do not change with the incubation time within the range studied.

### Aggregation of Aβ42 – Influence of Aβ42 concentration

Next, we examined the influence of the Aβ42 concentration on the aggregation. As can be seen in Fig. 1b, the FCS curves shift to longer correlation times as the concentration of Aβ42 is increased, due to the growing contribution of slower aggregates. This is corroborated by the increase of the aggregates’ contribution (1-*R*) as the Aβ42 concentration increases, as shown in Fig. 1f. In contrast, the diffusion time of the aggregates, *τ*_*D2*_, does not show systematic changes as the Aβ42 concentration varies (Fig. 1d), but fluctuates randomly. These results indicate that the increase of the Aβ42 concentration leads to an increase in the number of the aggregates of Aβ42, but not to a change in their size. This behaviour is typical for cooperative, micelle-like aggregation where the aggregates are in equilibrium with the monomers. Micelle-like intermediates in Aβ fibril assembly were already reported by Yong *et al*. in SANS experiments for the smaller peptide Aβ40^16^.

Based on this experimental evidence, we have formulated a model for the composition and the properties of the samples under study. In the samples, monomers coexist with aggregates characterized by a mean aggregation number $\bar{n}$ (see Theory section in SI). The application of this model to the results of the FCS measurements allows us to determine the fraction of Aβ42 in solution which is aggregated (fraction of aggregated amyloid or degree of aggregation *γ*), as well as to estimate the parameters related to the size and conformation of the aggregates.

### Critical aggregation concentration

**Figure 2:**
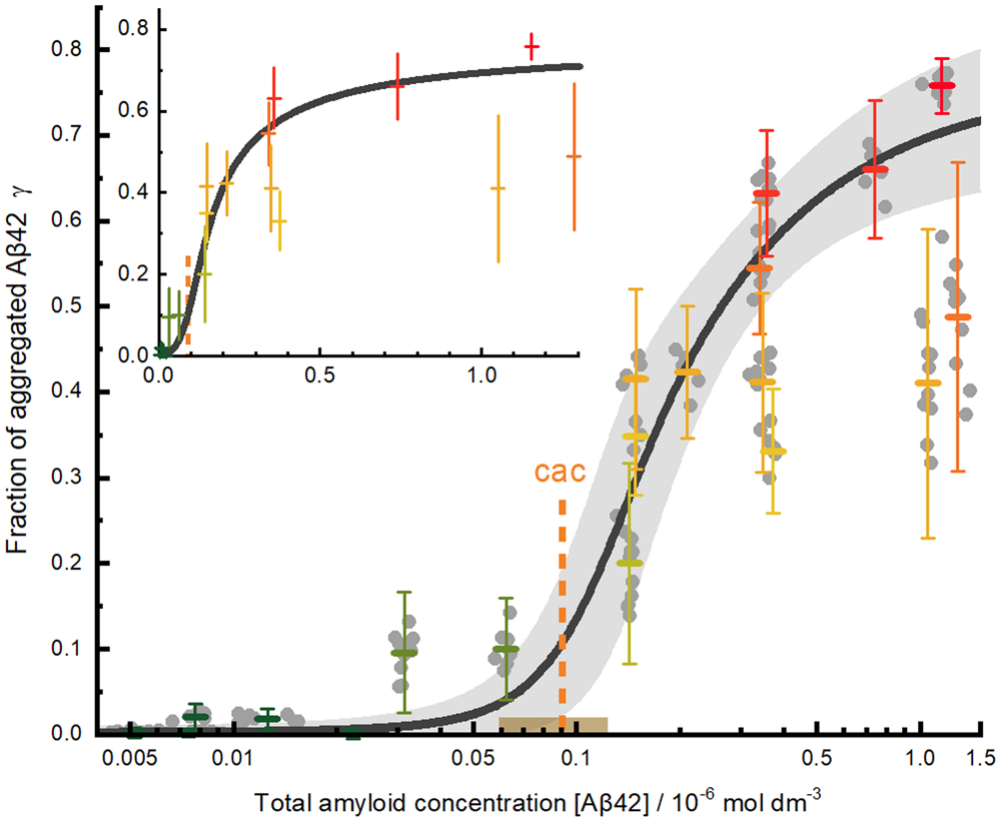
Fraction of aggregated Aβ42, *γ*, against the total Aβ42 concentration in solution. The small grey dots are the individual *γ-*values of all samples at different incubation times obtained following the analysis procedure described in the SI. The coloured horizontal bars represent the mean value of each sample. The error bars correspond to three standard deviations of the individual values of each sample. The black solid line is the result of a weighted fit of the aggregation model (Supplementary Equation S36) to the mean values with a *cac* of 0.09 μM (vertical orange dashed line) and a width of the transition region ± *r*·*cac* = ±33 nM (shaded interval around the *cac*). Note that the weighted fit is mainly defined by the samples with a low standard deviation. Main Panel: logarithmic concentration scale. Inset: mean values and fit on a linear concentration scale. The Aβ42 concentration refers to the experimentally determined real concentrations in solution.

For each FCS curve, we calculated the experimental value of the degree of aggregation *γ* by applying the data analysis procedure described in the SI. Figure 2 shows the degree of aggregation *γ* determined from all the measurements performed at different incubation times (grey dots in Fig. 2), plotted against the total Aβ42 concentration in solution. The Aβ42 concentration in solution was estimated for each sample and incubation time from the FCS data as described in the SI. The fraction of aggregated amyloid *γ* is negligible at Aβ42 concentrations lower than 0.02 μM, but it increases sharply at concentrations around 0.1 μM and stabilizes at higher concentrations.

This critical-concentration dependence of the degree of aggregation is typical for a spontaneous cooperative aggregation process such as, for example, the self-assembly of surfactants into micelles. In these processes, at low concentrations, only monomers are present in solution. However, when the concentration exceeds a critical aggregation concentration (*cac*), aggregates form abruptly. Above the *cac*, the number of aggregates increases, but not their size, which remains within a narrow distribution. In order to extract quantitatively a value of *γ* we applied a fit model developed in our group for the self-assembly of surfactants^30,31^. We fitted this model to the mean *γ*-value of each sample (coloured horizontal bars in Fig. 2), weighted by the standard deviation of the values at different incubation times. In spite of the strong fluctuations and bias of some of the *γ*-values, the confidence interval of the fitted curve overlaps with all but one of the error bars. From this weighted fit, we obtain a value of *cac* = 91 ± 14 nM for Aβ42 *in vitro* under physiological conditions and a transition concentration interval of σ = *r*·*cac* = 33 nM (Fig. 2, see SI for further details on the fit). These results indicate that under our experimental conditions the transition from monomeric to aggregated Aβ42 occurs in a concentration interval between 60 nM and 120 nM.

To our knowledge, these are the first experimental data reported for the degree of the early aggregation of Aβ42. They provide direct experimental evidence for the existence of a critical concentration for the early aggregation of this amyloid. Moreover, the fact that these data follow the model for surfactant self-assembly suggests that the early aggregation of Aβ42 shows an analogous micelle-like cooperative behaviour. The model allows for a quantitative estimation of the *cac* of Aβ42.

The value we obtain for the *cac* of Aβ42 is of the same order as other values reported for this amyloid, despite disparate experimental conditions and measuring techniques. It is half the reported limit of 0.2 μM below which aggregates could not be detected using thioflavin T as a fluorescent probe^19^, a result that evidences the higher sensitivity of FCS for this kind of studies. Iljina *et al*. recently reported *cac* values of Aβ40 and Aβ42 using a sample preparation and an analysis method different to ours^20^. They estimated one value of the *cac* of 28 nM from the concentration of soluble Aβ42 species in equilibrium with fibrils. They also present a figure with Aβ42 oligomer concentrations as a function of initial monomer concentrations (Fig. 2b in that work) and indicate the Aβ42 concentration at which the oligomer concentration ceases to increase as a second estimation of the *cac* (≈85 nM, taken from that figure). These data show a similar tendency to those in our Fig. 2 and could probably also be fitted with our aggregation model with a *cac* between 50–70 nM. Our *cac* value of about 90 nm is in better agreement with their estimation taken from the oligomer concentrations than with that from the fibril supernatant. This agreement supports the interpretation that this *cac* corresponds to the formation of oligomers in the early aggregation steps.

### Size and conformation of the aggregates

Our detailed quantitative model also allows us to characterize the aggregates regarding their size and conformation. From the relationship between the diffusion times of the aggregates and the experimental (uncorrected) mean aggregation numbers, we obtain an exponent ν = 0.75 for the molar mass dependence of the diffusion coefficient of the aggregates, *D*~*M*^−ν^, (step 4 of Data analysis procedure in SI and Supplementary Figure S2). This result indicates that the early aggregates of Aβ42 have an elongated structure, in very good agreement with the reported cylindrical geometry of Aβ40 micellar assemblies^16^.

**Figure 3:**
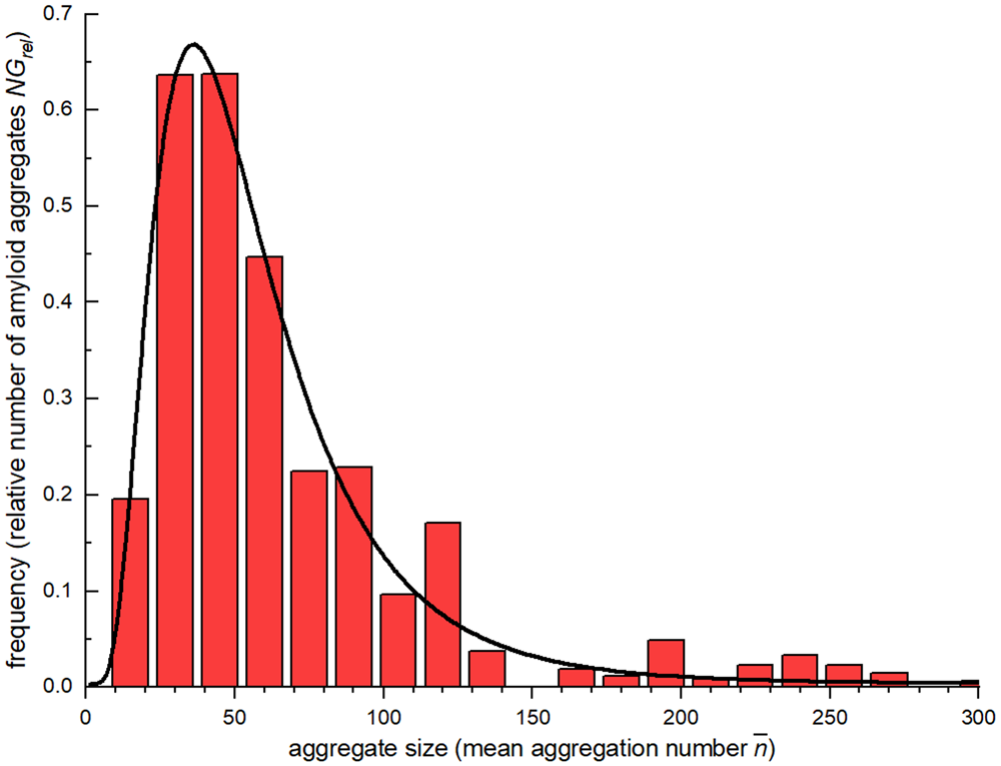
Size-distribution of early Aβ42 aggregates. Histogram of the aggregate sizes of Aβ42 representing the relative number of aggregates $N{G}_{rel}(\bar{n})$ with a mean aggregation number $\bar{n}$ observed in each FCS experiment at different Aβ42 concentrations and incubation times. The continuous curve is a fit of a log-normal distribution with a mean of $\bar{n}$ = 50 and a 68.3% interval of [28,88].

Furthermore, the size distribution of the early Aβ42 aggregates can be estimated based on the corrected mean aggregation numbers (see steps 5 and 7 of the Data Analysis Procedure in the SI). Figure 3 shows the histogram of the relative number of aggregates against their corrected mean aggregation numbers. This distribution can be well described by a log-normal curve, typical of naturally occurring growth processes. From the fit of this curve to the experimental data, we obtain a mean aggregation number of 50 Aβ42 molecules per aggregate, with most (68%) of the aggregates formed by 28 to 88 monomers. This corresponds to a mean hydrodynamic radius of the aggregates between 7.3 nm and 10.7 nm. These results are in accordance with those obtained for the smaller peptide Aβ40^16^, where micelle-like assemblies of 30–50 monomers and elongated geometries of around 7 nm hydrodynamic radius were detected. For Aβ42, we obtain slightly higher values of the aggregation number and hydrodynamic radius, as expected for this longer peptide. Those authors also observed no concentration dependence of the size or the conformation of the aggregates. However, our results differ from the much smaller sizes reported by other groups for Aβ42 oligomers^20,28,32^. This disagreement can be attributed to the presence of small proportions of a highly disaggregating solvent in the samples used in those works that could be expected to have an influence on the aggregation process. It must be noted that differences in the sample preparation protocols or in the conditions used for the aggregation study (especially temperature and salt concentration) can lead to disparate types of oligomers and fibrils^33^.

## Conclusions

Our results show that Aβ42 forms aggregates of 28–88 monomers when the monomer concentration exceeds a certain concentration in solution. This aggregate size does not change with time or with Aβ42 concentration within the ranges studied. These findings provide direct experimental evidence that early Aβ42 aggregation follows a micelle-like cooperative process. This process would be the primary nucleation pathway for the formation of oligomers that may behave as nuclei for a further slow aggregation into fibrils^29^. Analysis of the degree of aggregation using a model describing micelle formation allows us to determine quantitatively the critical aggregation concentration as *cac* = 90 nM for the early Aβ42 aggregation *in vitro* under physiological conditions. The transition from monomeric to aggregated Aβ42 occurs in a critical nanomolar concentration interval between 60 nM and 120 nM.

The use of FCS has allowed us to observe the behaviour and to estimate the concentration only of the dissolved amyloid peptide despite its high adsorption to the interfaces. The big difference between the real and the nominal Aβ42 concentrations in *in vitro* experiments may explain the higher *cac*-values given by those studies that refer to the nominal concentrations.

The recently proposed surface-mediated aggregation pathway^24^ seems not to be effective below the critical concentration regime, but it may contribute to aggregation above the *cac*. Our results do not provide further information about this contribution. The role of this pathway should be evaluated in further studies.

The observed high molecular weight oligomers with an elongated geometry would correspond to the reported protofibrillar aggregates, which are mainly responsible for the neurotoxic damage^2^. Recent studies show that the degree of toxicity of amyloid oligomers depends both on their size and on their conformation, especially on the solvent-exposed hydrophobicity of the aggregates^7,13,33–35^. Regarding the size, the oligomers observed in this study would not have the highest toxicity according to previous studies^13^, but we have no information about their hydrophobicity.

We think that the results obtained in this study *in vitro* reveal essential features of the early aggregation of Amyloid-β (1–42) in aqueous solution and give estimates of the parameters involved, which could also be relevant *in vivo*, yielding pivotal information to understand the mechanisms regulating neurodegenerative diseases.

## Methods

### Preparation of monomeric Aβ42

For the disaggregation of the amyloid peptides, the protocols reported by Stine *et al*. were followed^36,37^. The commercial peptides (unlabelled Amyloid- β(1–42) from GenScript USA Inc. and HiLyte Fluor 488-β-amyloid(1-42) from AnaSpec Inc.) were treated with hexafluoroisopropanol (HFIP) in order to break down the potential aggregates present in solution. The amyloid was dissolved in HFIP at a concentration of about 1 mg/ml and the solution was incubated during 1 h with occasional mixing and then shaken for about 20 min. Next, the solution was split up into several vials and the HFIP was evaporated under a stream of nitrogen. The vials were transferred into a desiccator and vacuum was applied during 3 h to remove the remaining traces of HFIP. The resulting dried aliquots of monomeric Aβ42 were sealed and stored at −20 °C.

### Sample preparation

To study the early aggregation process, the samples were freshly prepared from the previously disaggregated monomeric amyloid. The peptide was first suspended in anhydrous dimethyl sulfoxide (DMSO) to a concentration of ≈1 mM and shaken for about 15 min in order to dissolve it. Then a certain volume of PBS buffer (pH 7.2, 150 mM NaCl) was added to obtain the desired final concentration of amyloid. The solution was gently stirred for 1–2 min prior to use. For samples with both labelled and unlabelled amyloid, instead of PBS buffer, solutions with labelled amyloid were used for the dilution of the unlabelled peptide. The potential effect of the DMSO on the aggregation process was not considered, as no systematic variations of the aggregated amount were observed for samples with similar amyloid concentrations and different percentages of DMSO.

For this study, samples were prepared with a fixed nominal concentration (0.3 μM) of the fluorescently labelled Amyloid-β (1–42) (Aβ* and different concentrations of unlabelled Amyloid-β (1–42) (Aβ°), nominally in the range from 0.1 μM to 45 μM. In addition, samples containing only the labelled amyloid were prepared in order to characterize the monomeric Aβ42 and to check that the starting samples were properly disaggregated.

Due to the strong adsorption of Aβ42 to the interfaces, only a fraction of the initial nominal concentration was finally available in the sample solutions. Nevertheless, the real concentration of dissolved Aβ42 could be estimated for each sample from the FCS measurements (see Theory section in SI). If not otherwise indicated, all Aβ42 concentration indicated throughout this study refer to these experimentally determined real concentrations in solution.

### Measurement of FCS curves

For each sample, Fluorescence Correlation Spectroscopy (FCS) measurements were performed as a function of the time after the dissolution of the peptide (incubation time). About 100 μl of the sample were placed into a well of a 96-well glass-bottom microplate (Whatman Ltd.) and left about 5 min to achieve a constant temperature (25 °C) and to equilibrate. Usually a significant decrease of intensity was observed during this time, which is attributed to the adsorption of the amyloid to the interfaces.

The FCS setup has been described before^38,39^ and is summarized in the SI. Power series with samples containing only Aβ* and mixtures of Aβ* and Aβ° were performed in order to select a suitable excitation power for the FCS measurements that avoids distortions due to photobleaching and optical saturation. These measurements were also used for the characterization of the monomeric Aβ42 (see SI for details).

### Data analysis

FCS curves were fitted using Supplementary Equation S1 and S2 to obtain the diffusion correlation times of monomers and aggregates and their contributions to the diffusion term. Further data analysis was performed based on a model describing the composition and properties of the samples in order to extract quantitative information on the aggregation process and the size and conformation of the aggregates. This model is explained in detail in the Theory section of the SI, as well as the derived data analysis procedure. Parameter uncertainties indicate standard errors.

### Determination of translational diffusion coefficients

The translational diffusion coefficients were estimated from the diffusion correlation times as $D={w}_{xy}^{2}/4{\tau }_{D}$, calibrating the radial width *w*_*xy*_ of the sample volume with Rhodamine 123 as reference dye with known value^40,41^ of *D*_ref_ = (5.0 ± 0.1) × 10^−10^ m^2^ s^−1^. Under the conditions used in this work Rhodamine 123 has a mean diffusion time of *τ*_*D*_ = 41.3 ± 0.2 μs, corresponding to a radial width of *w*_*xy*_ = 0.29 μm. The diffusion coefficients are given for 25 °C.

### Data availability

The datasets generated during and/or analysed during the current study are available from the corresponding author on reasonable request.

## Electronic supplementary material

Supplementary Information
