# Supplementary material for: Critical aggregation concentration for the formation of early Amyloid-β (1–42) oligomers
Source: Sci Rep. 2018 Jan 29;8:1783. doi: 10.1038/s41598-018-19961-3 (PMC5789034; doi:10.1038/s41598-018-19961-3)
Supplement: Supplementary file 1 — Supplementary Information [file 41598_2018_19961_MOESM1_ESM.pdf]

SUPPLEMENTARY INFORMATION

Critical aggregation concentration for the formation of early Amyloid-β (1-42) oligomers

Mercedes Novo, Sonia Freire, and Wajih Al-Soufi*

Department of Physical Chemistry, Faculty of Science, Campus Lugo

University of Santiago de Compostela, Spain

E-mail: m.novo@usc.es

Contents

FCS measurements 2
FCS setup 2
Analysis of FCS curves 2
FCS Power series 3
Theory 4
Abbreviations 4
Assumptions 4
General Equations 5
Estimation of the mean aggregation number from the diffusion times 7
Fraction of aggregated amyloid 7
Total Amyloid Concentration in Solution 8
Micelle-Model for the aggregation of amyloid around the critical aggregation concentration (*cac*) 9
Data analysis procedure 9
Fit of the degree of aggregation 12
References 13

FCS measurements

FCS setup

The FCS setup has been described before[1, 2]. The sample was excited through a microscope objective (Olympus, UPLSAPO 60xW/1.20, water immersion) by a diode laser at 489 nm (Becker&Hickl, BDL-485-SMC). The emitted fluorescence was collected by the same objective, passed through a pinhole (Thorlabs, ∅=50 µm, US), filtered by a band-pass filter (Semrock, Brightline HC 525/45, US) and then split into two beams by a nonpolarizing beamsplitter cube (Newport, 05BC17MB.1, US). Each beam was then focused onto an avalanche photodiode (MPD50CTC APD, ∅=50 µm, MPD, Italy). The detector signals were processed and stored by two TCSPC-modules (SPC 132, Becker & Hickl GmbH, Berlin, Germany). Typically 10-20 million photons were collected for each correlation curve. All measurements were made at stabilized temperature, 25.0 ± 0.5ºC.

Analysis of FCS curves

Amyloid monomers bind dynamically to the amyloid aggregates. This aggregation is much slower than the typical diffusion time of the monomers or aggregates across the FCS sample volume (slow exchange regime). Therefore, free and aggregated labelled amyloid is detected in FCS as two distinct species, each with its characteristic diffusion correlation time.

The FCS correlation curves were fitted with correlation functions for the translational diffusion of one (eq. (1)) or two (eq. (2)) independent species across the sample volume and an additional term due to the transitions of the dye to the dark triplet state[3-5]:

$$G_D = \frac{1}{\mathrm{N}}\left(1+\frac{\tau}{\tau_D}\right)^{-1}\left(1+\frac{\tau}{\omega^2\tau_D}\right)^{-\frac{1}{2}}\left(1+A_T e^{-\tau/\tau_T}\right) \tag{1}$$

$$G_D = \frac{1}{\mathrm{N}}\left(R\left(1+\frac{\tau}{\tau_{D1}}\right)^{-1}\left(1+\frac{\tau}{\omega^2\tau_{D1}}\right)^{-\frac{1}{2}}+\left(1-R\right)\left(1+\frac{\tau}{\tau_{D2}}\right)^{-1}\left(1+\frac{\tau}{\omega^2\tau_{D2}}\right)^{-\frac{1}{2}}\right)\left(1+A_T e^{-\tau/\tau_T}\right) \tag{2}$$

Here $\tau_{Di} = w_{xy}^2 / 4D_i$ is the diffusion correlation time of species *i* across the sample volume $V = \pi^{3/2} w_{xy}^2 w_z$ with aspect ratio $\omega = w_z / w_{xy}$. D_i is the translational diffusion coefficient of species *i*.

N is the apparent number of particles. It depends on the average number of particles of type 1 and 2 in the sampling volume $N_i = V C_i$, and on the brightness Q_i of these particles, defined by the product of the extinction coefficients, fluorescence quantum yields and detection efficiency $Q_i = \varepsilon_i \, \phi_{(F)i} \, g_i$:

$$\frac{1}{N} = \frac{Q_1^2 N_1 + Q_2^2 N_2}{\left(Q_1 N_1 + Q_2 N_2\right)^2} \tag{3}$$

The factors *R* and *1-R* give the relative contributions to the amplitude of species *1* and *2* respectively:

$$R = \frac{Q_1^2 N_1}{Q_1^2 N_1 + Q_2^2 N_2} \tag{4}$$

FCS Power series

FCS curves were measured for a sample of Aβ* in PBS buffer at different power values of the excitation radiation between 10 μW and 300 μW (Figure 1S). The plots of the diffusion times, τ_D, and the fluorescent counts per molecule, *cpm,* against power or irradiance show constant and linear behaviours, respectively, in the range from 10 μW to 100 μW and deviate only at higher power values due to the effect of photobleaching and optical saturation. When adding unlabelled amyloid Aβ° in order to provoke aggregation, fluorescence intensity increases linearly with the power up to 100 μW, indicating the same linear range as for the monomers (note that neither the τ_D of the aggregates nor the *cpm* can be used in this case due to the fluctuations in the size of the aggregates and the apparent number of molecules obtained for these mixtures, respectively). From these results, an excitation power of *P* = 80 μW was chosen for all FCS measurements [6], corresponding to a mean irradiance $I_0 / 2 = (2P)/(\pi w_{xy}^2)$ = 44 kW cm^{-2}.

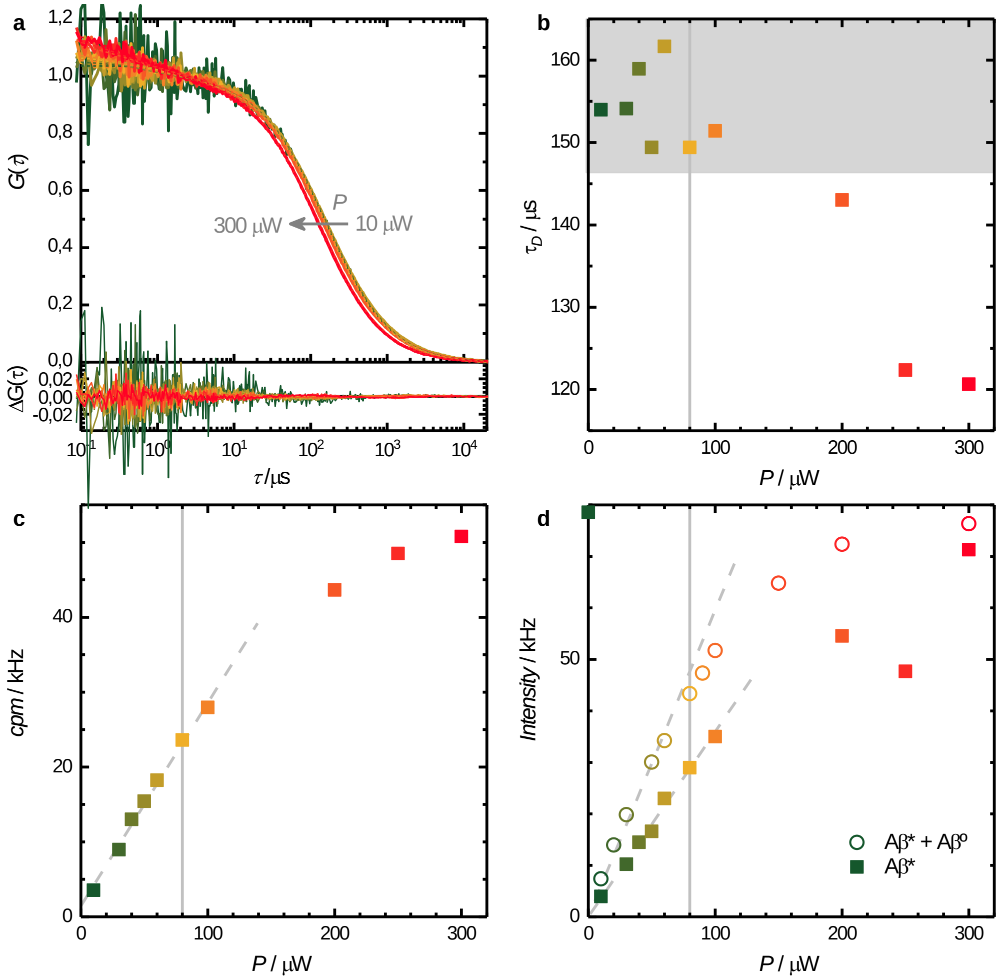

Figure S1: FCS-Power series. Panel a: normalized correlation curves of a sample of Aβ* in PBS buffer at different power values of the excitation radiation between 10 μW and 300 μW. Lines are the fits of the correlation function (eq. 1) to the experimental curves. Panel b: diffusion correlation times for different excitation powers from the fits in panel a. Panel c: fluorescent counts per second and molecule, *cpm*, obtained for the measurements of the curves in panel a. Panel d: fluorescence intensity obtained during the measurements of the curves of panel a (Aβ* in PBS buffer) and after the addition of unlabelled Aβ42 to the sample. The vertical grey lines indicate the excitation power of 80 μW used in all further measurements.

Theory

In order to extract quantitative information about the early amyloid aggregation we first establish the equations describing the species and their properties as observed in the experimental data. Then we describe the data analysis procedure applied.

Abbreviations

Species:

A	Amyloid (in any form, unlabelled or labelled, free or aggregated)
A°, A^{*}	Unlabelled and labelled amyloid (free or aggregated)
A_f°, A_f^{*}	Free (monomeric) amyloid, unlabelled and labelled
A_g°, A_g^{*}	Aggregated amyloid, unlabelled and labelled
G, G°, G^{*}, G^{i}	Amyloid aggregate, any form, unlabelled, labelled and *i*-times labelled

Numbers:

NX, NX_0, NX_T	Number of species X, at any moment, initial and total
NG	Number of aggregates

Properties:

$\bar{n}$	Mean number of amyloid per aggregate (*mean aggregation number*)
$\bar{i}$	Mean number of labelled amyloid per aggregate (*mean occupation number*)
$\bar{i}^{*}$	Mean number of labelled amyloid per *labelled* aggregate
γ	Fraction of aggregated amyloid (*degree of aggregation*)
α	fraction of labelled amyloid of the total amyloid, $\alpha = [A^{*}]/[A]$
cac	critical aggregation concentration
r	relative transition width around the *cac* (micelle model)

FCS parameter:

$\tau_{D1} \equiv \tau(A_f^{*})$	diffusion time of free labelled amyloid
$\tau_{D2} \equiv \tau(G)$	diffusion time of amyloid aggregates
$N_1 \equiv NA_f^{*}$	number of free labelled amyloid monomers per FCS sample volume
$N_2 \equiv NG^{*}$	number of labelled amyloid aggregates per FCS sample volume
$Q_1 \equiv Q_f$	brightness of a free labelled amyloid
$Q_2 \equiv Q_{G^*}$	mean brightness of labelled amyloid aggregates
Q_g	brightness of a labelled amyloid molecule in an aggregate
$q = Q_g/Q_f$	brightness ratio between a free and an aggregated labelled amyloid
I	registered total fluorescence intensity (photon counts per second)
R, 1-R	relative contributions of the free amyloid and the aggregates to the full amplitude of the diffusion term
$cpm = I / N$	photon counts per second and molecule

Assumptions

Based on the experimental evidences presented in the main text we apply the following assumptions:

A1 The mean $\overline{n}$ of the size-distribution of the amyloid aggregates does not depend on the incubation time or on the concentration.

A2 The mean number of labelled amyloid per (labelled) aggregate, occupation number $\overline{i}$ ($\overline{i}^*$), follows a Poisson distribution.

A3 The labeling has neither effect on the aggregation properties of the amyloid monomer nor on its adsorption affinity. This implies that the fraction *α* of labelled amyloid is the same for free and for aggregated amyloid, as well as for adsorbed and dissolved amyloid.

A4 The brightness Q_g of a labelled amyloid molecule in an aggregate is independent of the size $\overline{n}$ or the occupation i of the aggregate.

General Equations

FCS yields absolute numbers of particles (dye-labelled monomers or aggregates) observed in the sample volume. Therefore, we express the following equations in "numbers" (per volume) instead of concentrations $[\mathrm{X}] = NX / V$.

The total number of amyloid (*NA*) is the sum of free unlabelled (NA_f°), free labelled (NA_f^*), aggregated unlabelled (NA_g°), and aggregated labelled amyloid (NA_g^*):

$$NA = NA_f^\circ + NA_f^* + NA_g^\circ + NA_g^* \tag{5}$$

Note that *NA* refers to the available amyloid in the solution as determined from the FCS measurements.

The *labelled fraction α* of the total amyloid is

$$\alpha = \frac{NA^*}{NA} = \frac{NA_f^* + NA_g^*}{NA_f^\circ + NA_f^* + NA_g^\circ + NA_g^*} \tag{6}$$

Following assumption A3, the fraction *α* of labelled amyloid does not depend on the aggregation, and is the same for free and aggregated amyloid:

$$\frac{NA_f^*}{NA_f^\circ + NA_f^*} = \frac{NA_g^*}{NA_g^\circ + NA_g^*} = \frac{NA^*}{NA} = \alpha \tag{7}$$

This leads to other useful relations:

$$NA_f^\circ = \left(\frac{1}{\alpha} - 1\right) NA_f^*, \qquad NA_g^\circ = \left(\frac{1}{\alpha} - 1\right) NA_g^*, \qquad NA = \frac{NA_f^* + NA_g^*}{\alpha} \tag{8}$$

The fraction of aggregated amyloid (*degree of aggregation*) ***γ*** can be determined from the labelled amyloid alone:

$$\gamma \equiv \frac{NA_g}{NA} = \frac{NA_g^\circ + NA_g^*}{NA} = \frac{(1/\alpha - 1)NA_g^* + NA_g^*}{(NA_f^* + NA_g^*)/\alpha} = \frac{NA_g^*}{NA_f^* + NA_g^*} \tag{9}$$

The *mean number of amyloid per aggregate* (*size*, or *aggregation number*) is given by the number of aggregated amyloid and the number of aggregates:

$$\overline{n} = \frac{NA_g}{NG} \tag{10}$$

The labelled amyloid is randomly distributed among the aggregates, leading to aggregates that bear none, one, or multiple labels. The mean number of labelled amyloid per aggregate, *mean occupation number* $\bar{i}$, is given by the product between the labeling-fraction *α* and the mean aggregation number $\bar{n}$:

$$\bar{i} = \frac{NA_g^*}{NG} = \frac{NA_g^*}{(NA_g^\circ + NA_g^*)/\bar{n}} = \alpha \cdot \bar{n} \tag{11}$$

The probability that an aggregate contains *i* labelled amyloids is given by a Poisson distribution (assumption A3):

$$P(i) = \frac{NG^i}{NG} = \frac{\bar{i}^i e^{-\bar{i}}}{i!} \tag{12}$$

The number of unlabelled (NG° , *i* = 0) and labelled (NG^* , *i* > 0) aggregates is then given by

$$\begin{aligned} NG^i &= NG \cdot P(i) \\ NG^\circ &= NG \cdot P(0) = NG \cdot e^{-\bar{i}} \\ NG^* &= NG \cdot P(i > 0) = NG \cdot (1 - P(0)) = NG \cdot (1 - e^{-\bar{i}}) \\ NG &= NG^\circ + NG^* \end{aligned} \tag{13}$$

With $NG = NA_g^* / \bar{i}$ (eq. (11)) and $\bar{i} = \alpha \cdot \bar{n}$ we obtain then

$$NG^* = \frac{NA_g^*}{\bar{i}} \cdot (1 - e^{-\bar{i}}) = \frac{NA_g^*}{\alpha \cdot \bar{n}} \cdot (1 - e^{-\alpha \cdot \bar{n}}) \tag{14}$$

High labelling fractions $(\alpha \to 1)$ lead to high occupation numbers $\bar{i} >> 1$ so that all aggregates contain at least one labelled amyloid $(NG^\circ \to 0)$. Then we get

$$NG^* \approx NG = \frac{NA_g^*}{\bar{i}} \quad (\bar{i} >> 1) \tag{15}$$

In the limit that all amyloid is labelled $(\alpha = 1)$ we obtain of course

$$\bar{i} = \bar{n}, \quad NG = NG^* = \frac{NA_g^*}{\bar{n}} \quad (\alpha = 1) \tag{16}$$

The *brightness of labelled amyloid* may change upon aggregation. According to the assumption A4 the ratio *q* between the brightness of a free (Q_f) and an aggregated (Q_g) labelled amyloid molecule is independent $\bar{n}$ or i (note that *q* refers to a single labelled amyloid molecule):

$$q = \frac{Q_g}{Q_f} \tag{17}$$

In order to estimate the mean brightness $Q_2 \equiv Q_{G^*}$ of labelled aggregates from the brightness of a *single* aggregated labelled amyloid, Q_g, one needs to know the *mean number of labelled amyloid per* labelled *aggregate* $\bar{i}^*$:

$$Q_2 \equiv Q_{G^*} = \bar{i}^* Q_g \tag{18}$$

This occupation number $\bar{i}^*$ is different from the mean occupation $\bar{i}$, which counts also unlabelled aggregates. Using eq. (14) we get:

$$\bar{i} = \frac{NA_g^*}{NG} = \alpha\bar{n}$$
$$\bar{i}^* = \frac{NA_g^*}{NG^*} = \bar{i}\left(1-e^{-\bar{i}}\right)^{-1} = \alpha\bar{n}\left(1-e^{-\alpha\bar{n}}\right)^{-1} \tag{19}$$

In the limits of very high or very low occupation numbers we get as expected:

$$\bar{i}^* \approx \bar{i} \qquad (\bar{i} >> 1)$$
$$\bar{i}^* \approx 1 \qquad (\bar{i} << 1) \tag{20}$$

The ratio between the mean brightness of the labelled aggregates and the brightness of the free amyloid is then:

$$\frac{Q_2}{Q_1} \equiv \frac{Q_{G^*}}{Q_f} = q\bar{i}^* = q\frac{\bar{i}}{1-e^{-\bar{i}}} = q\frac{\alpha\bar{n}}{1-e^{-\alpha\bar{n}}} \tag{21}$$

Equation (21) relates the mean aggregation number $\bar{n}$ to the brightness of labelled free amyloid and aggregates.

With eq. (9) the degree of aggregation γ can then be estimated from N_1 and N_2 determined in FCS:

$$\left.\begin{array}{r} NA_f^* \equiv N_1 \\ NA_g^* = NG^* \cdot \bar{i}^* = N_2 \cdot \bar{i}^* \end{array}\right\} \gamma = \frac{NA_g^*}{NA_f^* + NA_g^*} = \frac{N_2 \cdot \bar{i}^*}{N_1 + N_2 \cdot \bar{i}^*} \tag{22}$$

Estimation of the mean aggregation number from the diffusion times

The diffusion times of homogeneous particles across the FCS sample volume change with their molar mass following a power law, where the exponent υ is related to the geometry of the particle[7].

$$\tau_D = \frac{w_{xy}^2}{4 \cdot D} = \frac{w_{xy}^2}{4 \cdot a \cdot M^{-\upsilon}} = b \cdot M^{\upsilon} \tag{23}$$

The mean molar mass of the aggregates M_2 is given by the mean aggregation number and the molar mass of the amyloid monomers M_1 (disregarding the mass of the labels):

$$M_2 = \bar{n} \cdot M_1 \tag{24}$$

The ratio of the diffusion times of aggregates and monomers is then

$$\frac{\tau_{D,2}}{\tau_{D,1}} = \frac{b_2 M_2^{\upsilon_2}}{b_1 M_1^{\upsilon_1}} = \frac{b_2 M_1^{\upsilon_2} \bar{n}^{\upsilon_2}}{b_1 M_1^{\upsilon_1}} = \frac{b_2}{b_1} M_1^{(\upsilon_2-\upsilon_1)} \bar{n}^{\upsilon_2} \tag{25}$$

$$\log\frac{\tau_{D,2}}{\tau_{D,1}} = \log\left[\frac{b_2}{b_1} M_1^{(\upsilon_2-\upsilon_1)}\right] + \upsilon_2 \log\bar{n} \tag{26}$$

The slope of the linear relationship given in eq. (26) υ_2 gives information about the geometry of the aggregates.

Fraction of aggregated amyloid

In FCS only labelled aggregates contribute to the signal. From equations (1) - (4) follows:

$$N_1 = \frac{Q_2^{\,2} N R}{\left(Q_1(1-R) + Q_2 R\right)^2}, \qquad N_2 = \frac{Q_1^{\,2} N (1-R)}{\left(Q_1(1-R) + Q_2 R\right)^2} \tag{27}$$

The number of labelled amyloid aggregates $N_2 \equiv NG^*$ determined from eq. (27) does not depend on the occupation number *i* of the aggregates.

The registered fluorescence intensity *I* is given by

$$I = I_1 + I_2 = Q_1 N_1 + Q_2 N_2 \qquad (28)$$

Then the number of free labelled amyloid can be determined as

$$N_1 = \frac{R}{N}\frac{I^2}{Q_1^2} \qquad (29)$$

and the number and brightness of labelled aggregates are given by

$$N_2 = \frac{I - Q_1 N_1}{Q_2} \qquad (30)$$

$$Q_2 = \frac{1-R}{R}\frac{Q_1^2 N_1}{I - Q_1 N_1} \qquad (31)$$

The fraction of aggregated amyloid γ (*degree of aggregation*) can be estimated from the FCS-parameter *R*, avoiding the use of the absolute intensities detected in FCS since they bear great uncertainties. For this we reorder the definition of *R* (eq. (4)) and substitute eq. (18) and (19):

$$R = \frac{1}{1 + \dfrac{{Q_2}^2 N_2}{{Q_1}^2 N_1}} = \frac{1}{1 + \overline{i}^* q^2 \dfrac{NA_g^*}{NA_f^*}} \quad \Rightarrow \quad \frac{NA_f^*}{NA_g^*} = \overline{i}^* q^2 \frac{R}{1-R} \qquad (32)$$

Substitution in eq. (9) yields a relation between γ and *R*.

$$\gamma = \frac{NA_g^*}{NA_f^* + NA_g^*} = \frac{1-R}{1 + R(\overline{i}^* q^2 - 1)} \qquad (33)$$

Total Amyloid Concentration in Solution

With eq. (6) and (22) the total number of amyloid in the sample volume (FCS focus) is obtained as

$$NA = \frac{NA^*}{\alpha} = \frac{N_1 + N_2 \cdot \overline{i}^*}{\alpha} \qquad (34)$$

The total (dissolved) amyloid concentration is then obtained with the sample volume *V* determined from measurements with the reference dye with known diffusion coefficient as described above (N_A: Avogadro number):

$$[\mathrm{A}] = \frac{NA}{N_A\, V} \qquad (35)$$

Micelle-Model for the aggregation of amyloid around the critical aggregation concentration (*cac*)

The concentration of free and aggregated amyloid around the *cac* can be fitted with a model developed for the formation of micelles around the critical micelle concentration[8, 9]. With this model the degree of aggregation γ can be expressed as a function of the total amyloid concentration as:

$$\gamma = \frac{[\mathrm{A_g}]}{[\mathrm{A}]} = 1 - \frac{[\mathrm{A_f}]}{[\mathrm{A}]}$$
$$[\mathrm{A_f}] = cac\left\{1 - \frac{a}{2}\left[\sqrt{\frac{2}{\pi}}\, r\, \mathrm{e}^{-\frac{1}{2r^2}\left(\frac{[\mathrm{A}]}{cac}-1\right)^2} + \left(\frac{[\mathrm{A}]}{cac}-1\right)\left(\mathrm{erf}\left[\frac{1}{\sqrt{2}r}\left(\frac{[\mathrm{A}]}{cac}-1\right)\right]-1\right)\right]\right\} \qquad (36)$$
$$a = 2\left(1 + \sqrt{\frac{2}{\pi}}\, r\, \mathrm{e}^{-\frac{1}{2r^2}} + \mathrm{erf}\left[\frac{1}{\sqrt{2}r}\right]\right)^{-1}$$

The parameter r is the relative width of the transition region between free and aggregated amyloid around the *cac*[8].

Data analysis procedure

The following procedure was applied to the raw FCS curves measured with different amyloid concentrations in order to obtain the results presented in this contribution:

Step 1: Fit the FCS curves with the correlation functions

Determine the brightness Q_1 of free labelled amyloid from FCS experiments with monomeric labelled amyloid at low concentrations, fitting the one species model, eq. (1).

Fit each of the FCS curves of the experiments with different amyloid concentrations with the two species model of eq. (2) in order to obtain values for N, R, and τ_{D1} and τ_{D2}. From N, r, the detected intensity I, and equations (29) - (31) calculate the values of Q_2, N_1 and N_2. This set of data is further analysed in the following steps.

Step 2: Determine the brightness ratio q

Estimate the brightness ratio q from fits of eq. (21) to the experimental ratios Q_2/Q_1 as a function of the fraction of labelled amyloid α. Use experiments with low labelling fraction ($\alpha < 0.1$) which correspond to a mean number of labels per aggregate $\bar{i}^*$ near one. This yields a value of $q = 0.60 \pm 0.14$. The value of the mean aggregation number $\bar{n}$ determined from these fits has a very high uncertainty and is not used in the further analysis.

Step 3: Estimate uncorrected aggregation numbers $\bar{n}_u$

With the value of q determined in step 2 and the individual values of Q_1 and Q_2 calculate for each experiment the mean number of labels per aggregate $\bar{i}^* = Q_2/(qQ_1)$. Then estimate for each experiment a first uncorrected aggregation number $\bar{n}_u$ solving numerically $\bar{i}^* = \alpha\bar{n}/(1-e^{-\alpha\bar{n}})$ (eq.(19)). These values of the aggregation number $\bar{n}_u$ determined from the brightness ratios are strongly affected by the fluctuations

in the total intensities. They will be used in the next step but will then be substituted by better estimates derived from the diffusion times.

Step 4: Estimate the molar mass dependence of the diffusion times

Represent in a double logarithmic plot the ratios of the diffusion times τ_{D2}/τ_{D1} versus the aggregation numbers $\bar{n}_u$ (Figure 2S). From a global fit of the linear dependence (eq. (26)) to all experiments a mean slope of υ_2 = 0.75 ± 0.06 is obtained.

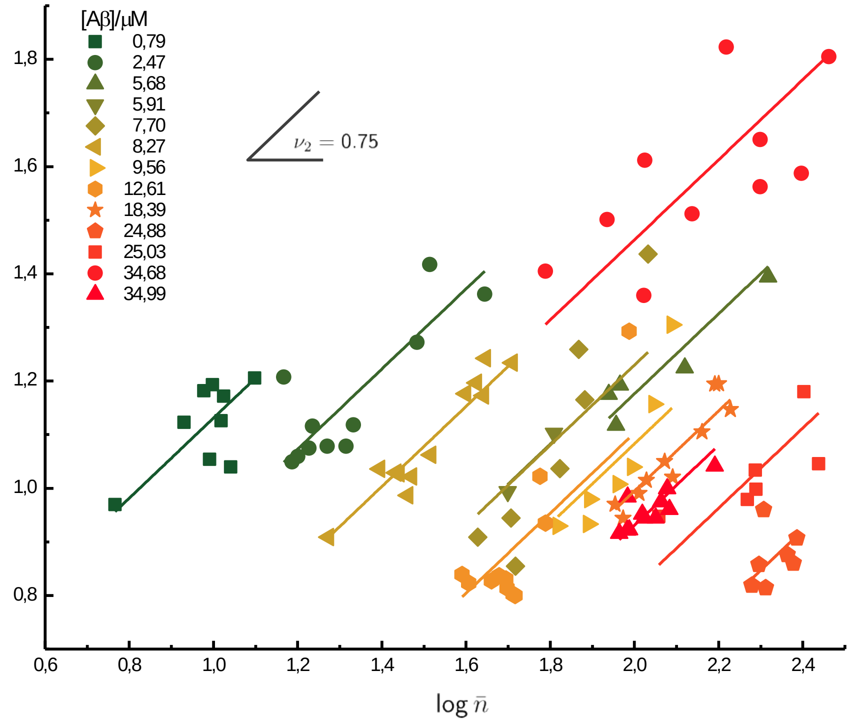

Figure S2: Double logarithmic plot of the ratios of the diffusion times τ_{D2}/τ_{D1} of Aβ versus the aggregation numbers $\bar{n}$ obtained from repetitive measurements of samples of different Aβ-concentrations. The straight lines are the result of a global linear fit with shared slope and individual intercepts. The indicated Aβ concentrations are nominal values.

Step 5: Estimate corrected aggregation numbers $\bar{n}_c$

From the ratios of the diffusion times calculate for each experiment new corrected aggregation numbers $\bar{n}_c$ from eq. (25), using the global fit result of step 4 for υ_2 and the weighted mean intercept of -0.31 ± 0.18.

Step 6: Determine the concentration dependence of the degree of aggregation γ

For each experiment estimate the mean number of labelled amyloid per labelled aggregate $\bar{i}^*$ from eq. (19) using the values of $\bar{n}_c$ determined before. With these values of $\bar{i}^*$, and *q* and *R*, calculate the degree of aggregation γ for each experiment from eq. (33).

Estimate the total available amyloid *NA* in each of the samples from eq. (34) and the amyloid concentration [A] from eq. (35).

Determine the critical aggregation concentration *cac* and the relative transition width *r* fitting the dependence of γ on the total amyloid concentration with the micelle-model of eq. (36).

Step 7: Calculate the distribution of the aggregate sizes

Construct the size-distribution from the mean number of aggregates $NG(\overline{n}_c)$ observed in each experiment with a mean aggregation number $\overline{n}_c$.

For this, first, get better estimates of the number of *labelled* aggregates $NG^*(\overline{n})$ in each measurement from the diffusion time data, calculating the brightness of the aggregates $Q_2 \equiv Q_{G^*} = \overline{i}^* \cdot q \cdot Q_f$ and then their number $N_2 \equiv NG^*(\overline{n})$ with eq. (27). The mean number of aggregates (labelled or not) can then be estimated as $NG(\overline{n}) = NG^*(\overline{n}) / (1 - e^{-\alpha \overline{n}})$ (eq. (13)).

Finally, the different amyloid concentrations used in each particular experiment have to be taken into account. For this divide the number of aggregates $NG(\overline{n})$ of each experiment by the number of aggregated amyloid $NA_g = \gamma NA$ expected at the given number of amyloids *NA* in the sample volume. This is the *relative number of aggregates* $NG_{rel}(\overline{n})$:

$$NG_{rel}(\overline{n}) = \frac{NG(\overline{n})}{NA_g} = \frac{NG(\overline{n})}{\gamma NA} \tag{37}$$

For the construction of the distribution curve sum up the values of $NG_{rel}(\overline{n})$ at discrete intervals of the aggregation numbers $\overline{n}$ of the individual experiments.

Finally, fit the distribution with a log-normal function. This fit yields the mean aggregation number of the distribution of $\overline{n}$ = 49.8 with a 68.3% interval of [28,88] for $\overline{n}$ [10].

Fit of the degree of aggregation γ

In Figure 2 of the main article we estimate the critical aggregation concentration *cac* from a fit of the degree of aggregation γ as a function of the Aβ42 concentration in solution. This figure presents the full experimental data set for the degree of aggregation γ. The data are the result of a long and tedious process to establish a reproducible protocol and to measure each sample with multiple repetitions. In spite of these efforts, the samples are still affected by minute variations during their preparation and handling, which inevitably lead to fluctuations and some bias in the determination of γ.

We finally obtained γ-values from 23 samples, each measured at 5 to 12 different incubation times, in total about 160 values. The following Figure S3 shows the unweighted fit of the surfactant aggregation model to these individual γ-values.

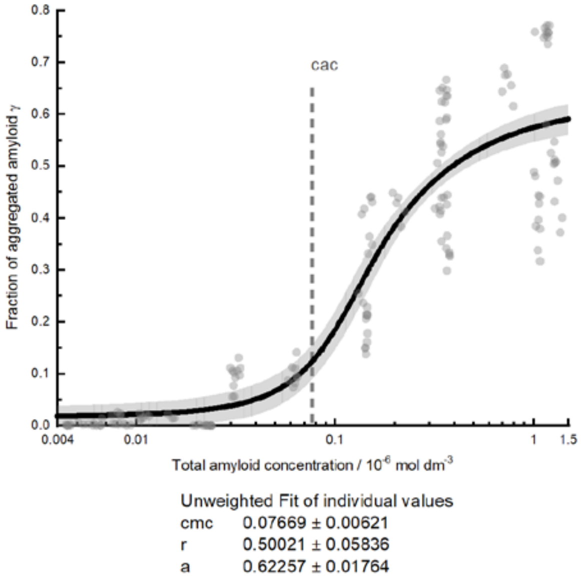

Figure S3: Unweighted fit of the individual γ-values shown in Figure 2 of the main article.

The model follows well the tendency in the data and gives a *cac* of about 77 nM. However, this fit treats the values of each of the samples measured at different incubation times as if they were statistically independent measurements. This is not true, because they share a common concentration and preparation bias. This leads to estimated uncertainties that are unrealistically small. Moreover, samples measured more often (at a higher number of incubation times) have a higher weight in this fit and distort the result.

From a statistical point of view, it seems to be better to represent each sample by the mean and the standard deviation of the γ-values of the different incubation times and then to fit these mean values weighted by their standard deviations. This fit gives those samples with a tighter standard deviation a much higher weight (σ^{-2}) and thus reduces the influence of samples with stronger fluctuations. This weighted fit yields in our opinion the best estimate for the *cac* using this fit model and is the one used in the article.

References

1. Bordello, J., Novo, M. & Al-Soufi, W. Exchange-Dynamics of a Neutral Hydrophobic Dye in Micellar Solutions Studied by Fluorescence Correlation Spectroscopy. *J. Colloid Interface Sci.* **345**, 369-376 (2010).

2. Granadero, D., Bordello, J., Pérez-Alvite, M. J., Novo, M. & Al-Soufi, W. Host-Guest Complexation Studied by Fluorescence Correlation Spectroscopy: Adamantane–Cyclodextrin Inclusion. *Int. J. Mol. Sci.* **11**, 173-188 (2010).

3. Elson, E. L. & Magde, D. Fluorescence correlation spectroscopy. I. Conceptual basis and theory. *Biopolymers* **13**, 1-27 (1974).

4. Lakowicz, J. R. in *Principles of Fluorescence Spectroscopy* (Springer, USA, 2006).

5. Widengren, J. in *Fluorescence correlation spectroscopy: theory and applications* (eds Rigler, R. & Elson, E. S.) 276-301 (Springer Verlag, Berlin, 2001).

6. Eggeling, C., Widengren, J., Rigler, R. & Seidel, C. A. M. Photobleaching of fluorescent dyes under conditions used for single-molecule detection: evidence of two-step photolysis. *Anal. Chem.* **70**, 2651-2659 (1998).

7. Wilkins, D. K. *et al*. Hydrodynamic radii of native and denatured proteins measured by pulse field gradient NMR techniques. *Biochemistry* **38**, 16424-16431 (1999).

8. Al-Soufi, W., Piñeiro, L. & Novo, M. A model for monomer and micellar concentrations in surfactant solutions: Application to conductivity, NMR, diffusion, and surface tension data. *J. Colloid Interface Sci.* **370**, 102-110 (2012).

9. Piñeiro, L., Freire, S., Bordello, J., Novo, M. & Al-Soufi, W. Dye exchange in micellar solutions. Quantitative analysis of bulk and single molecule fluorescence titrations. *Soft Matter* **9**, 10779-10790 (2013).

10. Limpert, E., Stahel, W. a. & Abbt, M. Log-normal Distributions across the Sciences: Keys and Clues. *Bioscience* **51**, 341-341 (2001).
